# Targeting inflammation to reduce cardiovascular disease risk: a realistic clinical prospect?

**DOI:** 10.1111/bph.13818

**Published:** 2017-06-10

**Authors:** Paul Welsh, Gianluca Grassia, Shani Botha, Naveed Sattar, Pasquale Maffia

**Affiliations:** ^1^ Institute of Cardiovascular and Medical Sciences, College of Medical, Veterinary and Life Sciences University of Glasgow Glasgow UK; ^2^ Centre for Immunobiology, Institute of Infection, Immunity and Inflammation, College of Medical, Veterinary and Life Sciences University of Glasgow Glasgow UK; ^3^ Hypertension in Africa Research Team (HART) North‐West University, Potchefstroom campus South Africa; ^4^ Department of Pharmacy University of Naples Federico II Naples Italy

## Abstract

Data from basic science experiments is overwhelmingly supportive of the causal role of immune‐inflammatory response(s) at the core of atherosclerosis, and therefore, the theoretical potential to manipulate the inflammatory response to prevent cardiovascular events. However, extrapolation to humans requires care and we still lack definitive evidence to show that interfering in immune‐inflammatory processes may safely lessen clinical atherosclerosis. In this review, we discuss key therapeutic targets in the treatment of vascular inflammation, placing basic research in a wider clinical perspective, as well as identifying outstanding questions.

**Linked Articles:**

This article is part of a themed section on Targeting Inflammation to Reduce Cardiovascular Disease Risk. To view the other articles in this section visit http://onlinelibrary.wiley.com/doi/10.1111/bph.v174.22/issuetoc and http://onlinelibrary.wiley.com/doi/10.1111/bcp.v82.4/issuetoc

**Abbreviations**
ApoB: apolipoprotein B
ApoE: apolipoprotein E
CANTOS: The Canakinumab Anti‐inflammatory Thrombosis Outcomes Study
CHD: coronary heart disease
CIRT: the Cardiovascular Inflammation Reduction Trial
CRP: C‐reactive protein
CVD: cardiovascular diseases
DCs: dendritic cells
ECs: endothelial cells
eNOS: endothelial NOS
GULO: L‐gulonolactone oxidase
HLA‐DR: human leukocyte antigen‐DR
HMG‐CoA: 3‐hydroxy 3‐methyl glutaryl CoA
ICAM‐1: intercellular adhesion molecule 1
IL‐1RA: IL‐1 receptor antagonist
LDL‐C: LDL‐cholesterol
LDLr: LDL receptor
Lp‐PLA_2_: lipoprotein‐associated PLA_2_
MI: myocardial infarction
oxLDL: oxidized LDL
RA: rheumatoid arthritis
RCTs: randomized controlled trials
SMCs: smooth muscle cells
TNFR: TNF receptor
VCAM‐1: vascular cell adhesion protein 1
WT: wild type

## Introduction

Atherosclerosis‐related cardiovascular diseases (CVD) are the leading cause of mortality worldwide (WHO, 2011). Immune responses play a decisive role in all phases of atherosclerosis (Galkina and Ley, 2009; Libby and Hansson, 2015), and inflammation contributes to plaque vulnerability (Hansson *et al.,* 2015). Atherosclerosis‐prone conditions accelerate immune cell recruitment into the arteries in the early and advanced stages of the pathology (Galkina *et al.,* 2006; Maffia *et al.,* 2007; Swirski *et al.,* 2016), and in experimental models, antigen‐presenting cell/T‐cell interactions have been shown in the arterial wall leading to local T‐cell activation and production of pro‐inflammatory cytokines (Koltsova *et al.,* 2012; Macritchie *et al.,* 2012; Sage *et al.,* 2014). In the advanced stages of the pathology, immune responses are tightly controlled *in situ* by the formation of artery tertiary lymphoid organs in the adventitial connective tissue adjoining arteries. These lymphocyte aggregates control primary T‐cell responses while bypassing secondary lymphoid organs exerting a protective effect on atherosclerosis in mice (Hu *et al.,* 2015; Srikakulapu *et al.,* 2016). Therefore, there is a range of inflammatory processes underpinning atherogenesis, which might be amenable to interventions.

Data from observational epidemiological studies also give some support to the inflammatory hypothesis of CVD. A host of prospective cohort data show that elevated circulating levels of C‐reactive protein (CRP), or indeed almost any other circulating inflammatory marker, are associated with an increased risk of future CVD events, even after adjusting for established classical CVD risk factors (Woodward *et al.,* 2007; Danesh *et al.,* 2008; Welsh *et al.,* 2011; Kaptoge *et al.,* 2012). These data suggest that low grade systemic inflammation precedes incident cardiovascular events and, as such, also imply that inflammation might cause vascular diseases that lead to major CVD events. Indeed, similar epidemiological associations between elevated cholesterol and blood pressure and risk of CVD have also been established (Lewington *et al.,* 2002; Sniderman *et al.,* 2011), and we know these to be causal risk factors due to supporting data from randomized controlled trials (RCTs) with specific pharmacological interventions [Cholesterol Treatment Trialists' (CTT) Collaborators *et al.,* 2012; Ettehad *et al.,* 2016]. However, although the association of these inflammatory biomarkers with CVD appears to be independent of other risk factors, and the utility of these biomarkers in clinical risk prediction warrants debate, experience tells us to be cautious with interpreting even strong associations as causal, given the potential for residual confounding.

**Figure 1:**
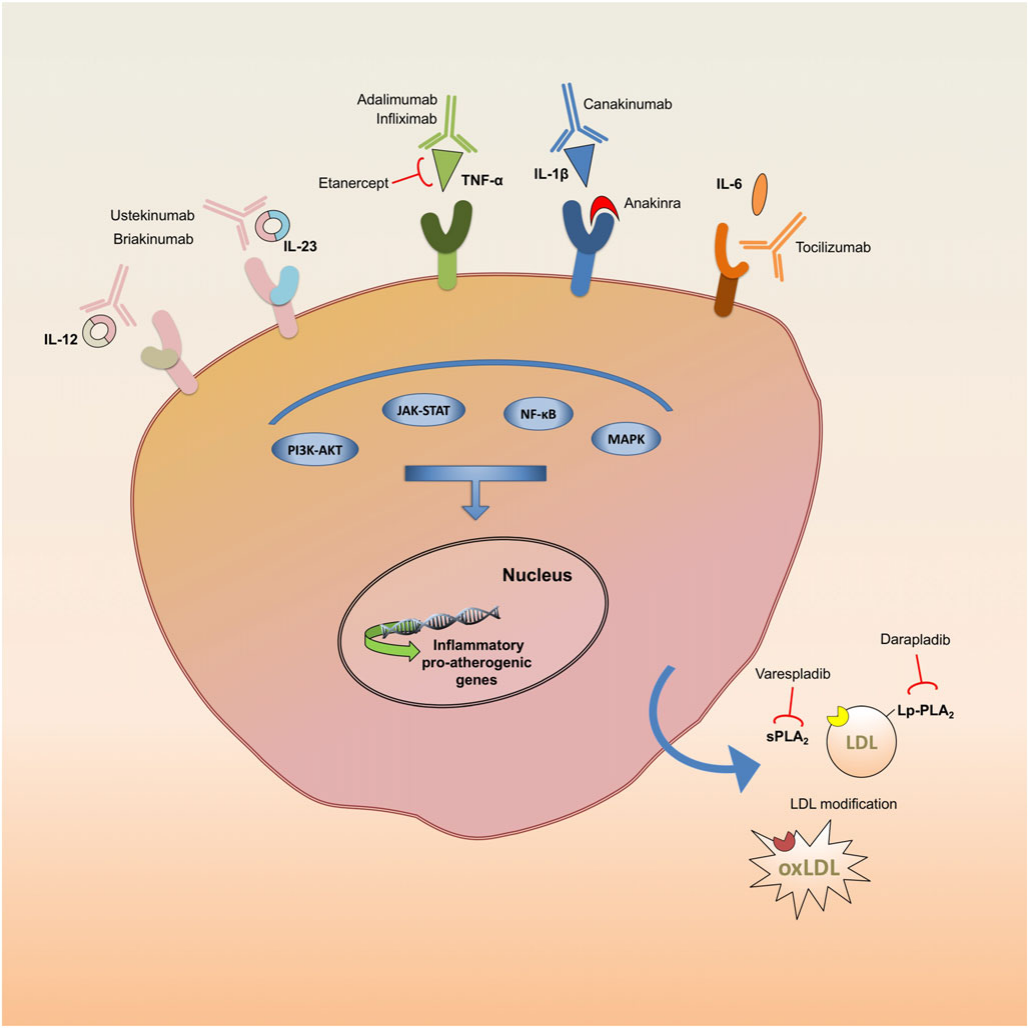
Pro‐atherogenic and inflammatory pathways targeted by prospective anti‐atherosclerotic antibodies and inhibitors.

In this review, we will discuss the key potential therapeutic targets in the treatment of vascular inflammation (Figure 1), placing basic research in to a wider clinical perspective, as well as identifying questions yet to be addressed.

## Lessons from the antioxidant vitamins

Claims about the efficacy of L‐ascorbic acid (vitamin C) in the prevention of the common cold, cancer, and CVD can be traced back to the influence of the double Nobel Laureate, Linus Pauling (Pauling, 1971). Indeed, there is an abundance of literature investigating the important issue of whether localized oxidative stress causes an inflammatory response in the vasculature, and other organs. Under this hypothesis, there may be a vicious positive feedback cycle between inflammation and oxidative stress, causing vascular remodelling and plaque development (Montezano *et al.,* 2015). In support of this idea, basic scientific experiments, and limited trial data suggest that vitamin C prevents free radical‐induced lipid peroxidation (Huang *et al.,* 2002). Vitamin C induces proliferation and concomitantly prevents *in vivo* apoptosis of endothelial cells (ECs), increasing the recovery of the endothelial layer following vascular damage (Rössig *et al.,* 2001; Saeed *et al.,* 2003). In addition, vitamin C is an essential regulator of collagen synthesis (Murad *et al.,* 1981; Davidson *et al.,* 1997; Qiao *et al.,* 2009). Both humans and guinea pigs are unable to synthetize vitamin C due to an inactivating mutation of the L‐gulonolactone oxidase (GULO), and a supplement of vitamin C in the diet is necessary otherwise the development of scurvy. Interestingly, chronic deprivation of vitamin C in diet, produce intimal lesions in guinea pigs (Willis, 1953). The corresponding KO strain, GULO^−/−^, fed a diet without vitamin C, showed extensive vascular impairment including disruption of elastin layers and desquamation of ECs (Maeda *et al.,* 2000). Apolipoprotein‐E deficient mice (apoE^−/−^), also lacking GULO showed a reduction of 40% in plaque collagen content (Nakata and Maeda, 2002), supporting a potential involvement of vitamin C in plaque stability rather than in plaque formation. It was therefore thought that vitamin C might ameliorate some of the downstream effects of an inflammatory response, and therefore prevent atherosclerosis.

**Figure 2:**
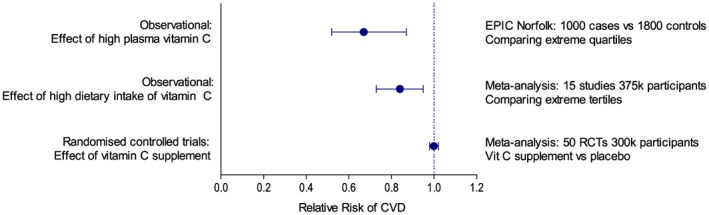
Summary of studies investigating the role of vitamin C in CVD risk. Data from Boekholdt *et al.* (2006), Ye and Song (2008) and Myung *et al.* (2013).

Indeed, observational studies suggest that low vitamin C levels might predict CVD outcomes. For example, in the EPIC‐Norfolk study, individuals in the highest quartile for plasma vitamin C had 33% lower risk of cardiovascular events (Boekholdt *et al.,* 2006). In a meta‐analysis of 15 studies (375 000 participants), those with the highest third of dietary intake of vitamin C were at 16% lower risk of CVD events (Ye and Song, 2008). Yet, despite these tantalizing data, meta‐analysis of 50 RCTs not only failed to show any benefit of supplementation with vitamin C in the prevention of CVD, but tight confidence intervals also essentially exclude any possibility of clinically meaningful benefits (Myung *et al.,* 2013) (Figure 2). This pattern, whereby basic science and observational data support a protective role of antioxidant vitamins in CVD, but trials of the relevant supplement provide no evidence to support the compelling and coherent theories, has been repeated for other antioxidants (Ye *et al.,* 2013). It could be argued that supplementations trials are more likely to fail when many of the participants are not ‘deficient’ in the vitamin in question. For example, 75% of participants in NHANES took the recommended daily intake of vitamin C (Fulgoni *et al.,* 2011). However, it is also worth noting that, similarly, many of the participants in other observational cohort studies will not be deficient in the studied vitamin. This does not appear to influence the observational association between vitamin status and outcome, and therefore does not offer a satisfactory explanation to resolve the apparent inconsistencies between observations studies and controlled trials. Further arguments about optimal delivery route, and dose of vitamin supplements in trial settings may be pertinent. However, it is worth noting that other pharmacological interventions, such as statins, have a ‘sliding scale’ of biological effects at a wide range of doses (Weng *et al.,* 2010); it is not clear why very specific doses of conventionally dietary antioxidants would be necessary to see any treatment benefit. Therefore, even the most rigorous approaches to data analysis struggle to overcome confounding and the reverse causality inherent in observational studies. While these data do not directly refute the inflammatory hypothesis of CVD, they do illustrate the caution always needed in interpreting observational associations as evidence of causality.

## Mendelian randomization studies

Mendelian randomization attempts to substantially attenuate or, in some cases, to eliminate the problems of confounding and reverse causality in classical observational epidemiology by exploiting the random allocation of genetic material at conception (Davey Smith and Hemani, 2014). Within a hypothetical population, one group of people has genetic variant(s) that lead to lower average circulating inflammatory markers over their life course, while the other group does not possess these variants. All other traits (such as adiposity, smoking and alcohol intake) should normally be equally distributed in the comparator groups. This situation can then be viewed as analogous to a RCT, and consequently, confers a level of stronger evidence of causality than classical epidemiology. Any differences in health outcomes between these two groups of people can be attributed to the concentration of inflammatory markers.

Strong evidence based on such data indicates that the inflammatory marker CRP does not cause CVD. In a meta‐analysis of nearly 200 000 participants, the relative risk for coronary heart disease (CHD) was 1.00 (0.90 to 1.13) per 1 SD higher genetically raised CRP concentration (Wensley *et al.,* 2011). Whether these polymorphisms might explain subtleties in the biology of atherosclerosis, such as the accumulation of monomeric CRP in plaques (Eisenhardt *et al.,* 2009), is not presently clear. In contrast however, genetic variants which lead to higher circulating concentrations of IL‐6 receptors (IL‐6R) (and consequently less IL‐6 cell signalling and lower circulating CRP) appear protective against CHD (IL6R Mendelian randomisation Consortium, 2012; Sarwar *et al.,* 2012). This has fuelled interest in the hypothesis that upstream key regulator cytokines are players in the development of atherosclerosis, and these two independent studies remain the most relevant findings to date supporting the inflammatory hypothesis.

## Lipid lowering therapies, inflammation and CVD

Statins, which act by inhibiting 3‐hydroxy 3‐methyl glutaryl CoA (HMG‐CoA) reductase, are the front‐line drug used for lipid reduction in primary and secondary CVD prevention. Their efficacy in reducing cholesterol and preventing CVD events is widely accepted on the basis of a large body of RCTs (CTT Collaborators *et al.,* 2012). However, basic science has published evidence over many years to suggest that statins might have pleiotropic effects (Bellosta *et al.,* 2000). Specifically of interest for this review, the idea that statins prevent CVD not only through lipid reduction but also through a lipid independent, anti‐inflammatory action has been extensively debated (Kinlay, 2007; Babelova *et al.,* 2013; Sirtori, 2014).

Statins can exert their putative pleiotropic effects through a broad range of mechanisms. They inhibit Rho‐GTPase isoprenylation through reducing geranyl‐geranylpyrophosphate production during cholesterol biosynthesis (Liao and Laufs, 2005; Cai *et al.,* 2015). This is leading to increased expression of endothelial NOS (eNOS) and NO production (Liao and Laufs, 2005). Statins have also been shown to increase NO production *via* activation of the PI3K‐Akt pathway through phosphorylation of Akt (Kureishi *et al.,* 2000). *In vivo*, a single dose of simvastatin to either wild type (WT) or apoE^−/−^ mice increased endothelium‐derived NO production (Scalia *et al.,* 2001). Moreover, statins can suppress the activity of pro‐oxidant enzymes (such as NADPH oxidase) in the endothelium (Margaritis *et al.,* 2014).

Statins also affect leukocyte trafficking at the inflammatory site. Atorvastatin, simvastatin and cerivastatin reduced the expression of intercellular adhesion molecule (ICAM)‐1 and lymphocyte function‐associated antigen‐1 on human ECs and circulating peripheral blood mononuclear cells stimulated with TNF‐α (Rezaie‐Majd *et al.,* 2003). Statins have also been shown to reduce, *in vitro*, in human primary cells, the production of the chemokine CCL2 (Romano *et al.,* 2000), and the secretion of matrix metalloproteinase (MMP)‐9 (Wong *et al.,* 2001; Wang *et al.,* 2016).

Statins can also directly affect the adaptive immune response. They have been shown to inhibit the inducible promoter IV of the transactivator CIITA in several cell types, such as the human ECs and monocyte/macrophages, and thereby repress MHC‐II mediated CD4^+^ T‐cell activation (Kwak *et al.,* 2000). Moreover, statins reduce the expression of CD40 in human vascular ECs, smooth muscle cells (SMCs), macrophages and fibroblasts (Mulhaupt *et al.,* 2003). Simvastatin and atorvastatin have been shown to reduce the expression of other costimulatory molecules such as CD83 and CD86 and human leukocyte antigen‐DR (HLA‐DR) induced by LPS in human monocyte‐derived dendritic cells (DCs) from healthy patients leading to a reduced capability of DCs to induce T‐cell activation, proliferation and Th1 differentiation (Yilmaz *et al.,* 2006). Atorvastatin concomitantly induces activation of STAT‐6 and inhibition of STAT‐4 phosphorylation, leading to secretion of Th2 cytokines (IL‐4, IL‐5 and IL‐10) and TGF‐β and suppression of Th1 cytokines (IL‐2, IL‐12, IFN‐γ and TNF‐α) (Youssef *et al.,* 2002). Lovastatin also increases the recruitment of regulatory T‐cells in inflamed sites. This effect is dependent on the expression of CCL1, a chemokine up‐regulated by statin administration (Mira *et al.,* 2008). More recently, statin‐loaded reconstituted HDL nanoparticles have been shown to inhibit atherosclerotic plaque inflammation in apoE^−/−^ mice, demonstrating that statins can selectively inhibit vascular inflammation *in situ*, directly in the diseased vessel wall, without any systemic effect such as lipid lowering (Duivenvoorden *et al.,* 2014).

There is therefore a body of evidence showing how statins might exert anti‐inflammatory effects, although one must always bear in mind the potential for publication bias whereby only positive studies fitting a prevailing hypothesis are published whereas negative studies are not easily published or not pushed towards publication in the first place. In epidemiological studies, statin treatment certainly does lower circulating levels of CRP in RCTs (Ridker *et al.,* 1999, 2009; Albert *et al.,* 2001; Sever *et al.,* 2012, 2013; Soedamah‐Muthu *et al.,* 2015), but the underlying mechanism, and whether apparently decreased systemic inflammation translates into a reduction of cardiovascular events is highly controversial. The JUPITER (Justification for the Use of statins in Prevention: an Intervention Trial Evaluating Rosuvastatin) trial suggested that the degree of CRP lowering on statin treatment may offer insight into CVD risk reduction beyond LDL‐cholesterol (LDL‐C) lowering (Ridker *et al.,* 2009). However, this analysis has not been reproduced in other trial data (Sever *et al.,* 2012, 2013; Soedamah‐Muthu *et al.,* 2015); the vast majority of benefit from statins is predictable from the extent of LDL reduction alone. One study level meta‐analysis suggested a strong correlation between change in LDL and change in CRP for a range of lipid lowering agents including statins, ezetimibe, niacin, fibrates and fish oils (*r* = 0.80) (Kinlay, 2007), perhaps suggesting that CRP lowering is directly or indirectly related to lipid lowering. More recently, however, the new class of lipid lowering drugs, proprotein convertase subtilisin/kexin type 9 inhibitors, have emerged and it appears these have little or no effect on CRP despite reducing circulating LDL by more than 50% (Blom *et al.,* 2014; Cannon *et al.,* 2015). Therefore, CRP reduction is not an inevitable consequence of LDL‐lowering.

The debate about potential pleiotropic effects of statins will continue. However, it is clear that it will be extremely difficult to tease apart lipid‐lowering effects from anti‐inflammatory effects and thus estimate their relative importance for CVD events. Statins studies alone will not prove or disprove the inflammatory hypothesis of CVD. It should be noted that the degree of LDL‐C reduction explains nearly all the CVD benefit seen in clinical trials of statins or indeed other agents so that one does not need to evoke alternative statin effects to explain benefits (Collins *et al.,* 2016).

## Autoimmune disease, biological agents and cardiovascular disease

It is well established that patients with a range of chronic systemic autoimmune conditions, including rheumatoid arthritis (RA), ankylosing spondylitis, psoriasis and irritable bowel disease, are at modestly increased risk of cardiovascular events, independent of other traditional risk factors (del Rincón *et al.,* 2001; Andersen and Jess, 2014; Ogdie *et al.,* 2015). The mechanisms by which cardiovascular risk is elevated in RA patients remains unproven, but the primary candidate pathway is that systemic inflammation drives vascular dysfunction and atherosclerosis (Sattar *et al.,* 2003). Indeed, data from the CORRONA database of nearly 25 000 RA patients, followed‐up for median 2.7 years, shows that those with low disease activity or in remission (and therefore with a lower burden of systemic inflammation) have an approximately 60% decrease in risk of CVD events, compared with those classified with high disease activity (Solomon *et al.,* 2015). Use of glucocorticoid treatment has long been a mainstay to reduce the pain and inflammation associated with RA, and it has been hypothesized that these anti‐inflammatory interventions might prevent CVD. Indeed, older studies in animal models suggest dexamethasone reduces atherosclerosis (Makheja *et al.,* 1989). However, the longer‐term side effects of steroid treatment, including potential causes of CVD, like diabetes, central obesity and hypertension, are likely to cause their own cardiovascular risks (Souverein *et al.,* 2004; Walker, 2007). Indeed, evidence from the RA field suggests high‐dose steroids have a net adverse association with CVD risk (Agca *et al.,* 2017).

The availability of a range of specific anti‐inflammatory interventions has revolutionized treatment of patients with RA and other autoimmune conditions; TNF‐α blockers and IL‐6 receptor blockers are now common and efficacious (if expensive) second or third line treatment options after use of conventional disease modifying anti‐rheumatic drugs. The availability of these and other biological agents has opened a world of possibilities with regards to testing the anti‐inflammatory hypothesis of CVD using a variety of different pathways.

## TNF‐**α** and IL‐6 in CVD

### Experimental data

Several lines of evidence support a pro‐atherogenic role for TNF‐α. TNF‐α binding to the TNF‐α receptors, TNFR1 or TNFR2, activates NF‐κB (Grassia *et al.,* 2010) and p38 MAPK (Sprague and Khalil, 2009) and therefore the transcription of proinflammatory genes including those for IL‐1β, IL‐8, CCL2, ICAM‐1, vascular cell adhesion protein (VCAM)‐1 and MMPs in a variety of cell types including lymphocytes, macrophage, ECs and vascular SMCs (Grassia *et al.,* 2009; Sprague and Khalil, 2009; Grassia *et al.,* 2010; Kalliolias and Ivashkiv, 2016). TNF‐α/apoE double knockout mice showed less atherosclerotic plaque formation compared to apoE^−/−^ mice (Brånén *et al.,* 2004; Ohta *et al.,* 2005), with reduced aortic expression of ICAM‐1, VCAM‐1, CCL2 as well as scavenger receptor class A (Ohta *et al.,* 2005). Treatment with TNF‐α binding protein reduced plaque development in apoE^−/−^ mice (Elhage *et al.,* 1998). Moreover, chimeric LDL receptor knockout (LDLr^−/−^) mice deficient in p55 TNF receptors (TNFR1) in bone marrow‐derived cells showed a reduction in atherosclerosis and reduced vascular recruitment of immune cells (Xanthoulea *et al.,* 2008). Several immune cells can be a source of TNF‐α in murine atherosclerotic vessels, including macrophages and the pro‐atherogenic B2 cell subset (Tay *et al.,* 2016). Importantly, TNF‐α can strongly influence plaque vulnerability. TNF‐α stimulates MMP production by SMCs as well as SMC activation, proliferation, and migration (Grassia *et al.,* 2009; Grassia *et al.,* 2010; Maddaluno *et al.,* 2012). Intriguingly, TNF‐α can concomitantly induce proliferation of human SMCs and apoptosis of ECs, confirming a scenario in which TNF‐α can alter the fibrous cap composition in the atheroma (Rastogi *et al.,* 2012).

Experimental data show a potential dual effect of IL‐6 on atherogenesis. *In vitro*, human macrophages stimulated with oxLDL produce IL‐6 (van Tits *et al.,* 2011), while stimulation with IL‐6 enhances the expression of adhesion molecules (ICAM‐1, VCAM‐1 and E‐selectin) in HUVEC (Watson *et al.,* 1996). IL‐6 mRNA is detectable in the aorta of apoE^−/−^ but not in WT mice (Sukovich *et al.,* 1998). The injection of recombinant IL‐6 increased lesion size in the aorta of apoE^−/−^ and C57Bl/6 mice fed a high‐fat diet and increased the expression of tissutal and circulating pro‐inflammatory cytokines (IL‐1β and TNF‐α) (Huber *et al.,* 1999). Moreover, treatment with a fusion protein of the natural IL‐6 trans‐signalling inhibitor soluble glycoprotein 130 reduced atherosclerosis in LDLr^−/−^ mice (Schuett *et al.,* 2012). In contrast, however, serum cholesterol levels and subsequent atherosclerotic lesion formation increased in apoE/IL‐6 double knockout mice compared to control animals, showing a less stable plaque phenotype and reduced circulation levels of IL‐10 (Schieffer *et al.,* 2004).

### Biological agents and hard CVD endpoints

Given the strong data on the role of these cytokines in atherosclerosis, the effect of blockade of these pathways on CVD risk in people with autoimmune disease is thus clearly of high interest. Despite this, RCTs of anti‐inflammatory therapies in RA patients and other inflammatory conditions have been powered to demonstrate improvements in disease activity rather than CVD endpoints, which requires far smaller sample sizes. Indeed, even meta‐analysis of randomized placebo controlled trials yields nowhere near sufficient power to investigate the effects of these biological agents on CVD events (Ryan *et al.,* 2011). Given that these drugs are now a cornerstone of treatment in RA patients, practicalities aside, a placebo controlled trial large enough to investigate the impact of these agents on CVD events is unfeasible due to the ethical implications of restricting some patients to placebo. The forthcoming ENTRACTE trial results (https://clinicaltrials.gov/ct2/show/NCT01331837), are highly anticipated and will directly compare the IL‐6 receptor blocker tocilizumab with the TNF‐α blocker etanercept in the prevention of CVD events in RA patients for the first time, but will not be able to test the inflammatory hypothesis directly. It is notable this study is powered only to rule out an upper hazard ratio of risk of 1.8 so it may not be powered sufficiently to provide a robust answer of CVD risk with tocilizumab, compared with that with etanercept.

Thus, given the lack of hard outcomes in trial data, the literature has turned to pharmaco‐epidemiological studies to investigate the effect of biological agents on CVD risk in patients with chronic autoimmune diseases. Meta‐analysis of observational studies and registries in RA, psoriasis and psoriatic arthritis patients suggest that those receiving TNF‐α blockers are at 30% lower risk (95% CI 0.54–0.90) of CVD than patients taking non‐biological therapies (Roubille *et al.,* 2015), perhaps offering some support to the notion that TNF‐α blockade is efficacious in reducing CVD events in people with systemic inflammatory conditions. However, pharmaco‐epidemiological studies are prone not only to confounding by established risk factors but also to confounding by indication. In the CORRONA database, for instance, patients taking TNF‐α blockers (compared to patients on non‐biological and non‐methotrexate based therapies) were at substantially lower risk of CVD events (HR 0.39, 95% CI 0.19 to 0.82) (Greenberg *et al.,* 2011). However, they were also slightly more likely to be female, were less likely to be tertiary educated, had higher scores on general health questionnaires, and were less likely to have had a previous myocardial infarction (MI). The authors adjust for these differences in many of the constituent studies of the meta‐analysis, but the fundamental problem remains that somewhere in the past an informed clinical judgement has been made; those prescribed biologics have fundamental differences in their characteristics from those not prescribed biologics, and these are impossible to fully measure, much less adjust for.

### Biological agents and surrogates of CVD

Further data on surrogate biomarkers of CVD in RA patients might be useful to infer the effects of administration of biological agents on CVD risk. The chronic inflammatory burden of RA patients depresses circulating total cholesterol and other lipid concentrations (Myasoedova *et al.,* 2010), a feature commonly seen in many chronic and acute inflammatory illnesses. Treatment with biological agents may be considered to ‘normalize’ total cholesterol, although some could nevertheless argue that an increase in cholesterol however achieved could still be potentially harmful. These drugs also have effects on several other pathways, as we recently demonstrated (Robertson *et al.,* 2013). In a *post hoc* study of the MEASURE trial of tocilizumab or placebo in 132 RA patients, total‐cholesterol, LDL‐C and triglyceride levels all increased in tocilizumab treated patients by week 12 (12.6, 28.1 and 10.6%, respectively), although there was no increase in small dense LDL or oxidized (ox)LDL (McInnes *et al.,* 2015). In addition, tocilizumab decreased lipoprotein (a) and decreased D‐dimer (McInnes *et al.,* 2015), a marker of thrombosis and fibrinolysis as well as CVD risk (Willeit *et al.,* 2013). Examination of downstream biomarkers of CVD may also be informative; for instance natriuretic peptides (such as N‐terminal pro B‐type natriuretic peptide, NT‐proBNP) are released during cardiac overload and are strong predictors of CVD risk (Welsh *et al.,* 2013, 2016a,b). Prospective data from an adalimumab (a TNF‐α blocker) treated cohort suggested that therapy reduced the cardiac biomarker and strong predictor of CVD risk, NT‐proBNP, but that study lacked a control arm (Peters *et al.,* 2010). However, this effect was not supported in a *post hoc* analysis of a RCT, where cardiac biomarkers were lowered by both tocilizumab and the standard care comparator (Welsh *et al.,* 2016a,b). Thus, the net effect of anti‐inflammatories on CVD risk is difficult to interpret from biomarkers alone.

### Safety profile of biological agents

Despite the efficacy of biological agents in chronic inflammatory conditions, their immunosuppressive properties have raised safety concerns, and consequently, they have been carefully evaluated in RCTs and by using registry data.

In a meta‐analysis of RCTs and open label studies, the TNFα blocker infliximab was associated with slightly more adverse events compared to placebo (OR 1.55, 95% CI 1.01–2.35), although this did not reach statistical significance for other biologics (Singh *et al.,* 2011). Non‐significant trends towards increases in pulmonary infections and tuberculosis reactivation were also noted. However, small numbers of incident malignancies preclude useful analyses and TNF‐α blockers are contraindicated in patients with heart failure. Attempts to use registry data for long‐term follow‐up of patients treated with biologics are likely to be subject to the same limitations described above.

Despite a predominantly encouraging safety profile in people with chronic diseases, there is a considerable ethical difference in giving immunosuppressive drugs to patients with chronic illness that may limit their quality of life (such as RA), and giving such drugs for the prevention of CVD, in which case the subclinical phase has little effect on quality of life, and a hard clinical event may never occur. For this reason, it is unlikely that the present generation of systemic anti‐inflammatory drugs will ever be prescribed in a primary prevention setting.

## PLA_2_

### Experimental data

The PLA_2_ superfamily are enzymes able to specifically hydrolyse fatty acids at the sn‐2 position of glycophospholipids releasing bioactive lipids, most importantly arachidonic acid and lysophospholipids. There are 15 different groups of PLA_2_ enzymes, each containing subgroups. Between them, the most studied in atherosclerosis include group II secretory PLA_2_ (sPLA_2_), and PAF acetylhydrolase, also known as lipoprotein‐associated (Lp)‐PLA_2_ (Burke and Dennis, 2009; Rosenson, 2010). Hydrolysis of membrane phospholipids by PLA2 is a key step in the production of precursors for eicosanoids and the potent inflammatory agent PAF. The development of sPLA_2_ inhibitors as possible anti‐inflammatory agents represents an interesting and active research field.

The proatherogenic activities of sPLA_2_ enzymes include the modification to circulating LDL, with conformational changes in apolipoprotein B (apoB)‐100 that impairs clearance by LDLr (Kleinman *et al.,* 1988). Thus, in mice overexpressing sPLA_2_, the time spent by LDL in the circulation increased, along with its susceptibility to oxidation and the loading of arterial macrophages with cholesterol (Ivandic *et al.,* 1999). Interestingly, phospholipid hydrolysis by sPLA_2_ causes conformational changes in apoB‐100 resulting in increased proteoglycan‐binding activity which facilitates LDL diffusion into the vessel wall (Flood *et al.,* 2004). In addition to these effects on LDL, the hydrolysis of phospholipids from cell membranes and lipoproteins increased local oxidative stress and levels of free arachidonic acid, lysophospholipids, and non‐esterified fatty acids (Rosenson and Hurt‐Camejo, 2012).

The relevance of sPLA_2_ isoforms in atherosclerosis has been investigated using knockout and transgenic mice. Transgenic mice expressing the human form of group IIa sPLA_2_ exhibited significant atherosclerotic lesions even when fed a low‐fat chow diet (Ivandic *et al.,* 1999). LDLr^−/−^ mice overexpressing group V sPLA_2_ by retrovirus‐mediated gene transfer showed increased atherosclerosis associated with collagen deposition in plaques (Bostrom *et al.,* 2007). Moreover, LDLr^−/−^ chimeric mice deficient in bone marrow group V sPLA_2_ showed less atherosclerosis compared to control animals (Bostrom *et al.,* 2007). On the contrary, the effect of group X sPLA_2_ seems to be protective. The overexpression of human group X sPLA_2_ in murine bone marrow cells of the LDLr^−/−^ chimeric mice leads to the reduction of Th1 response and to a 50% reduction of lesion formation (Ait‐Oufella *et al.,* 2013). All these results suggest different effects of the multiple sPLA_2_ isotypes and indicate the development of selective inhibitors of PLA_2_ isoforms as new possible compounds for the treatment of atherosclerosis.

Varespladib is an inhibitor of sPLA_2_ investigated in several animal models for its anti‐atherosclerotic effect. Treatment of apoE^−/−^ mice with varespladib resulted in the reduction of atherosclerosis development and a more stable plaque phenotype (Fraser *et al.,* 2009; Shaposhnik *et al.,* 2009). In atherosclerotic guinea pigs, treatment with varespladib was not effective in reducing atherosclerosis but decreased cholesterol accumulation in the aorta without changing serum cholesterol levels (Leite *et al.,* 2009).

Lipoprotein‐associated PLA_2_ (Lp‐PLA_2_) is an enzyme synthesized in macrophages and which travels in the circulation with LDL‐particles. Although the biology is controversial, several pieces of evidence suggest that Lp‐PLA_2_ is a pro‐inflammatory enzyme, due to its mediating role in the production of oxidized non‐essential fatty acids and lysophosphatidylcholine (Zalewski and Macphee, 2005), which are thought to be important in the recruitment and retention of inflammatory cells within plaques. Unexpectedly, overexpression of Lp‐PLA_2_ in apoE^−/−^ mice (Quarck *et al.,* 2001) or in balloon‐denuded rabbits (Turunen *et al.,* 2005) reduced endothelial damage and lesions formation. This anti‐atherogenic effect could, at least in part, be explained by the fact that Lp‐PLA_2_ hydrolyses PAF, a potent proinflammatory mediator (Talmud and Holmes, 2015).

Darapladib is a relatively selective inhibitor of Lp‐PLA_2_ (Rosenson and Hurt‐Camejo, 2012). Treatment of diabetic and hypercholesterolemic pigs with darapladib reduced development of advanced coronary atherosclerosis reducing the lyso‐PC content and necrotic core area of the lesion. Moreover, darapladib reduced the expression of several genes associated with macrophage and T lymphocyte activation in pig atherosclerotic vessels (Wilensky *et al.,* 2008), and CCL2, VCAM‐1 and TNF‐α in aortas from apoE^−/−^ mice (Wang *et al.,* 2011).

### Observational epidemiology and trial data

Observational epidemiology supports the notion that sPLA_2_ and LP‐PLA_2_ might be important pathophysiological pathways. Thus, higher circulating Lp‐PLA_2_ mass and activity was associated with increased CVD risk (Thompson *et al.,* 2010). In phase 2 trials, darapladib did exactly as predicted; it reduced IL‐6 by 12% and CRP by 13% (Mohler *et al.,* 2008), and also halted the progression of the necrotic core of atherosclerotic plaques (Serruys *et al.,* 2008). In contrast, the phase 3 STABILITY trial (15 828 CHD patients, 3.7 year follow‐up) and the SOLID‐TIMI 52 trial (13 026 ACS patients, 2.5 year follow‐up) disappointingly showed that darapladib did not reduce risk of a composite CVD endpoint (O'Donoghue *et al.,* 2014; White *et al.,* 2014). A similar lack of benefit was reported for varespladib (Nicholls *et al.,* 2014). Interestingly, Mendelian randomization predicted the findings for varespladib would show on benefit, ahead of the publication of the trial results (Holmes *et al.,* 2013).

## IL‐12 and IL‐23

### Experimental data

IL‐12 and IL‐23 are heterodimeric cytokines that share the subunit p40. The subunit p40 has been detected in foam‐cell‐like regions of the aortic plaque of apoE^−/−^ mice (Lee *et al.,* 1999). IL‐12 is expressed by lymphocytes, activated macrophages and DCs. IL‐12 activates the T‐bet transcription factor, leading to the up‐regulation of IFN‐γ production and polarisation of CD4^+^ T‐cells to the proinflammatory phenotype Th1 (Teng *et al.,* 2015). Recombinant IL‐12 accelerates the formation of atherosclerotic lesions in apoE^−/−^ mice (Lee *et al.,* 1999), while IL‐12 deficiency (IL‐12^−/−^/apoE^−/−^) resulted in reduced atherosclerosis (Davenport and Tipping, 2003). Interestingly, selective inhibition of IL‐12 production in macrophages led to a 50% decrease in aortic lesions in LDLr^−/−^ mice (Zhao *et al.,* 2002). Finally, blockade of IL‐12 by vaccination of LDLr^−/−^ mice resulted in a 60% reduction of atherosclerotic plaque, leading to a stable plaque phenotype (Hauer *et al.,* 2005).

The role of IL‐23 in atherosclerosis is poorly studied, despite the association observed between IL‐23 and disease progression in patients with carotid atherosclerosis. IL‐23 serum levels and the plaque mRNA expression levels were higher in patients with carotid atherosclerosis, compared with healthy patients (Abbas *et al.,* 2015).

The antibodies ustekinumab and briakinumab, which bind to the p40 subunit, were developed for the treatment of psoriasis. Given the arguments set out above, these biological agents might be expected to have a more direct anti‐inflammatory effect than PLA_2_ inhibitors.

### Trial data

Ustekinumab and briakinumab appear efficacious in reducing chronic inflammatory disease symptoms and perhaps reducing CRP (Toedter *et al.,* 2009; Strober *et al.,* 2011), but questions have been raised about safety, with a combined trial meta‐analysis reporting potentially increased major adverse cardiac events (OR = 4.23, 95% CI: 1.07–16.75, *P* = 0.04) (Tzellos *et al.,* 2013). The confidence intervals around this estimate are very large, and the nuances of quantifying the effect size lie in how the statistics for small event numbers in study arms are handled (sometimes no CVD events occurred).

The question remains as to whether these findings have any relevance for the inflammatory hypothesis of CVD. They certainly do illustrate the complex biology underlying atherogenesis and that an intervention that reduces inflammatory biomarkers cannot be presumed to be beneficial without hard endpoint data to support the findings (Table 1).

**Table 1:** Summary of studies investigating the effect of key inflammation‐related interventions on the risk for CVD and events

Target	Intervention	Observational studies (low weighted evidence of causal inference)	Mendelian randomization studies (intermediate weighted evidence of causal inference)	Randomized trials (strong weighted evidence of causal inference)
TNF‐α	Adalimumab	↑ Circulating TNF‐α ↑ CVD risk	NA	↑Infections (Singh *et al.,* 2011)
	Infliximab	Biological use ↓risk of CVD (Greenberg *et al.,* 2011)		**? CVD risk**
	Etanercept	Biological use ↓NT‐proBNP (Peters *et al.,* 2010)		
IL‐6R	Tocilizumab	↑ Circulating IL‐6 ↑CVD risk (Sarwar *et al.,* 2012; Interleukin‐6 Receptor Mendelian Randomisation Analysis (IL6R MR) Consortium, 2012)	IL‐6R SNPs rs7529229 and rs2228145 (Sarwar *et al.,* 2012) ↓CRP ↓**risk of CHD**	↑LDL‐C, ↓lipoprotein(a), ↓fibrinogen, ↓D‐dimer; ↔ small LDL, ↔ oxidized LDL, (McInnes *et al.,* 2015) **? CVD risk**
IL‐12/23 p40	Ustekinumab	NA	NA	↓CRP (Toedter *et al.,* 2009)
	Briakinumab			↑**CVD (**Tzellos *et al.,* 2013 **)**
IL‐1β	Canakinumab	NA	IL‐1Ra SNPs rs6743376 and rs1542176 (Interleukin 1 Genetics Consortium, 2015)	Ongoing (https://clinicaltrials.gov/ct2/show/NCT01327846)
IL‐1R	Anakinra (rIL‐1RA)		↓IL‐6; ↓CRP; ↑**risk of CHD**	
Lp‐PLA_2_	Darapladib	↑Lp‐PLA_2_ mass and activity ↑CVD risk (Thompson *et al.,* 2010)	Several SNPs including rs1051931 (Casas *et al.,* 2010) ↔ **risk of CVD**	↓IL‐6; ↓CRP (Mohler *et al.,* 2008) ↔ **risk of CVD** (O'Donoghue *et al.,* 2014; White *et al.,* 2014)
sPLA_2_	Varespladib	↑sPLA_2_ circulating concentration ↑CVD risk (Boekholdt *et al.,* 2005)	SNP rs11573156 (Holmes *et al.,* 2013) ↔ **risk of CVD**	↑**risk of CVD** (Nicholls *et al.,* 2014)
Multiple	Methotrexate	Methotrexate use ↓risk of CVD (Micha *et al.,* 2011)	NA	Ongoing (https://clinicaltrials.gov/ct2/show/NCT01594333)

NA, not applicable (note this may not mean there are no published studies, but that studies are comparatively small, prone to bias, or inconclusive); ↑, increase; ↓, decrease; ↔, unchanged; TC, total cholesterol; CAD, coronary artery disease; rIL‐1RA, recombinant IL‐1 receptor antagonist.

## Ongoing RCTs

The inflammatory hypothesis of CVD has, so far, never been directly tested in RCTs. Two major RCTs, powered for reduction in composite CVD endpoints will now formally test the inflammatory hypothesis in a secondary prevention setting. The Cardiovascular Inflammation Reduction Trial (CIRT: https://clinicaltrials.gov/ct2/show/NCT01594333) uses a methotrexate‐based intervention and the Canakinumab Anti‐inflammatory Thrombosis Outcomes Study uses a monoclonal antibody against IL‐β (CANTOS: https://clinicaltrials.gov/ct2/show/NCT01327846).

### Methotrexate experimental data

From a scientific perspective, the mechanisms by which methotrexate exerts anti‐inflammatory effects still need to be fully elucidated. Originally developed as an antifolate drug for the treatment of cancer, methotrexate inhibits cell division. It also shows a range of anti‐inflammatory mechanisms, independent of its antifolate activity, such as inhibition of T‐cell proliferation by affecting purine and pyrimidine metabolism, reduction of intracellular glutathione levels leading to reduced immune cell accumulation at inflammatory sites, and increased release of anti‐inflammatory adenosine (Cronstein, 2005). In TNF‐α‐stimulated human ECs, methotrexate down‐regulates pro‐inflammatory genes, such as those for TNF‐α, IL‐1β, CXCL2 and the toll‐like receptor 2, and up‐regulates the anti‐inflammatory TGF‐β1 gene (Bulgarelli *et al.,* 2012). Incubation with methotrexate prevents the conversion of lipid‐loaded THP‐1 cells into foam cells (Reiss *et al.,* 2008). Following treatment with methotrexate, adipose tissue from obese mice produced less proinflammatory (TNF‐α, IL‐6, leptin) and more anti‐inflammatory mediators (adiponectin and IL‐10) associated with reduced macrophage infiltration and inflammation (DeOliveira *et al.,* 2012). In addition, methotrexate down‐regulates the expression of adhesion molecules (ICAM‐1, E‐selectin, VCAM‐1) in human biopsies rich in inflammatory infiltrate (Dahlman‐Ghozlan *et al.,* 2004) and circulating levels of IL‐6 in psoriatic patients (Elango *et al.,* 2012). All these activities support a potential effect of methotrexate in the treatment of atherosclerosis. Indeed, methotrexate (4 mg·kg^−1^) intravenously injected four times a week for 30 days has been shown to reduce by 75% atherosclerosis formation in rabbits (Bulgarelli *et al.,* 2012). In addition, administration of methotrexate alone or in combination with etoposide carried in lipid nanoemulsion reduced macrophage, MMP‐9 and lesional content of proinflammatory cytokines, again in the atherosclerotic rabbit model (Bulgarelli *et al.,* 2013; Leite *et al.,* 2015).

### CIRT trial

CIRT randomizes low dose methotrexate (15–20 mg·week^−1^) plus folate (1 mg, 6 days week^−1^), versus placebo plus folate design. Participants include patients who have had a previous MI or multi‐vessel coronary artery disease, have type 2 diabetes and/or metabolic syndrome, and are therefore high‐risk, secondary prevention patients. As an intervention, methotrexate has several features to recommend it, including a long historical safety profile and very low cost. It should be noted that CIRT is powered to detect a 25% risk reduction in the methotrexate group; a considerable risk reduction against a background of gold standard secondary prevention therapies. If methotrexate fails to lower risk to this level, CIRT may not exclude the inflammatory hypothesis of CVD. The anticipated primary completion date of CIRT is presently the end of 2018.

### IL‐1 and CVD experimental data

IL‐1 is the first identified interleukin and affects virtually all cells and organs. It is the major pathogenic mediators of inflammatory and immune diseases (Garlanda *et al.,* 2013; Schett *et al.,* 2016). IL‐1α and IL‐1β share the same receptor (IL‐1R) and the same downstream signalling pathway. Instead, the IL‐1R antagonist (IL‐1RA) serves as a decoy receptor, inhibiting the effects of IL‐1. Both IL‐1α and IL‐1β are produced as precursors and activated by enzymic cleavage (Dinarello, 2011). IL‐1α mediates the early phases of sterile inflammation, whereas IL‐1β is produced as an inactive precursor from tissue‐resident macrophages and monocytes and is activated by caspase‐1. The system is also regulated upstream by the cleavage of procaspase‐1 by the NLRP3 inflammasome (Dinarello, 2011; Garlanda *et al.,* 2013). Neutrophils can also trigger IL‐1β response independently of caspase‐1 and inflammasome activation (Schett *et al.,* 2016).

The IL‐1 pathway seems to be an important player in atherosclerosis. IL‐1α and/or β induce the expression of ICAM‐1, E‐selectin and VCAM‐1 in HUVEC (Aziz and Wakefield, 1996), increasing adhesion of leukocytes (Bevilacqua *et al.,* 1985), leading to local amplification of innate and adaptive immunity (Garlanda *et al.,* 2013). IL‐1RA^−/−^ C57BL/6J mice fed a high cholesterol and cholate diet, developed foam cell lesions, whereas LDLr^−/−^ mice crossed with transgenic mice expressing high levels of murine sIL‐1RA, showed less atherosclerosis (Devlin *et al.,* 2002). The administration of human recombinant IL‐1RA in apoE^−/−^ mice also reduced plaque formation (Elhage *et al.,* 1998). On the contrary, apoE^−/−^ mice lacking IL‐1 receptor type I unexpectedly showed a vulnerable plaque phenotype including reduced SMC and collagen plaque content (Alexander *et al.,* 2012).

The specific role of the two IL‐1α and IL‐1β isoforms in atherosclerosis development is still under debate. Cholesterol crystals and oxLDL have been identified as endogenous triggers of the NLRP3 inflammasome, inducing the secretion of the active form of IL‐1β by plaque macrophages (Duewell *et al.,* 2010; Rajamäki *et al.,* 2010). This pathway is attractive as a potential explanation linking the phenotypes of elevated cholesterol, vascular inflammation and oxidative stress. IL‐1α and β have also been reported to enhance the expression of matrix enzymes (Schett *et al.,* 2016). In addition, deletion of IL‐1β (Kirii *et al.,* 2003) or the use of monoclonal antibodies against IL‐1β (Bhaskar *et al.,* 2011) inhibited the development of atherosclerosis in apoE^−/−^ mice. These findings may suggest a primary role for IL‐1β in the development of atherosclerosis. However, apoE^−/−^ mice lacking inflammasomes develop normal atherosclerotic lesions (Menu *et al.,* 2011) and, more importantly, fatty acid‐induced mitochondrial uncoupling abolished IL‐1β secretion, which turned the cholesterol crystal‐elicited response towards selective production of IL‐1α, as a potent inducer of vascular inflammation (Freigang *et al.,* 2013). This may be evidence of redundancy but could also suggest that IL‐1α could be targeted in patients with CVD. In summary, whether blockade of IL‐1β alone is sufficient to down‐regulate vascular inflammation still remains to be determined.

### CANTOS trial and epidemiological data of the IL‐1 pathway

Despite this optimism, Mendelian randomization data have provided some controversial findings. There are no established SNPs that can act as proxies for circulating IL‐β in Mendelian randomization studies. This is at least partly due to the lack of an assay sensitive enough to measure IL‐1β in healthy people. However, one recent study investigating genetic variants of IL‐1RA reported that variants associated with higher concentrations of IL‐1RA had lower concentrations of CRP (suggesting a true anti‐inflammatory effect), but were also puzzlingly associated with increased CHD (Interleukin 1 Genetics Consortium, 2015). As described previously, the IL‐1 cytokine superfamily signalling system and its regulation are complex (Herder and Donath, 2015). However, this study, and the widespread *post hoc* explanations of the data, really lays bare our ignorance of the pathways that underlie the inflammatory causes of CVD.

The randomized design in CANTOS, compares three arms of the IL‐1β blocker canakinumab (50, 150 and 300 mg administered subcutaneously every 3 months) to placebo. Participants in the trial are those who have experienced a recent MI and have a circulating hsCRP of >2 mg·L^−1^. CANTOS is powered for a 20% risk reduction in a composite CVD endpoint in any active arm compared to placebo, and combining doses will further improve power. The anticipated primary completion date is in late 2017 with presentation estimated for September.

## Conclusions and proposed next steps

This review highlights the conflict between observational epidemiology and animal models on the one hand, and disappointing Phase III trial results on the other. This conflict remains a major issue, and is one the main difficulties for the inflammatory hypothesis of CVD. There are important debates, outwith the scope of this review, on how to make animal models more relevant to human disease (Libby *et al.,* 2011), and also whether surrogate markers of CVD risk are truly useful to assess causality (Weintraub *et al.,* 2015).

Conduct of RCTs is a critical step in translating a wealth of biological information into tangible benefits for patients. If successful, the CANTOS and CIRT trials may provide the rationale for using anti‐cytokine‐based and anti‐inflammatory therapies for secondary prevention of atherosclerosis‐related CVD and may start a new era in the treatment of chronic vascular disorders. If unsuccessful, these trials will not conclusively disprove the inflammatory hypothesis of atherosclerosis, but might make conducting further trials in this area much more challenging.

Inflammation contributes to atherogenesis and disease development, and therefore, several other or combined anti‐inflammatory treatments may have the potential for preventing cardiovascular events. Importantly, evaluation of risks as well as benefits must drive the development of anti‐inflammatory treatments in CVD. Atherosclerosis is a life‐long process, and it is, therefore, unlikely that the present generation of systemic anti‐inflammatory drugs will ever be prescribed in a primary prevention setting, particularly given great gains in risk reduction in recent years with better treatments of blood pressure, cholesterol and population lowering of smoking rates *via* smoking bans.

From a biological perspective, several fundamental questions still need to be addressed. For instance, is atherosclerosis in humans a systemic or a local (vascular) immune disease? Are tertiary lymphoid organs in the adventitial connective tissue important in human pathology? The answers to this questions will pave the way for the design of more atherosclerosis‐specific treatments targeting directly vascular (rather than systemic) immune mechanisms for therapeutic utility and potentially reducing the risk of systemic immune suppression. Existing data highlight the complex nature of the immune system, and different signalling pathways may play different roles at different the stages of the pathology. Therefore, we may need different immunomodulatory treatments to affect disease initiation, progression, and/or plaque destabilisation and rupture. Targeting and inhibiting immune‐inflammatory response(s) may be crucial at the onset of the disease. On the contrary, enhancing atheroprotective immunity by expansion of regulatory T‐cells may be the best future therapeutic strategy in secondary prevention. Vaccination approaches have also been successful in experimental models. However, translation of these findings in clinical practice has only just started (Shah *et al.,* 2014; Kimura *et al.,* 2015).

Any new treatment will require robust safety evaluation and testing in randomized cardiovascular outcome trials well before potential adoption in clinical practice. As these studies progress, we will learn more about whether mechanisms of vascular inflammation are indeed viable diagnostic, prognostic and therapeutic targets in atherosclerosis.

### Nomenclature of targets and ligands

Key protein targets and ligands in this article are hyperlinked to corresponding entries in http://www.guidetopharmacology.org, the common portal for data from the IUPHAR/BPS Guide to PHARMACOLOGY (Southan *et al.,* 2016), and are permanently archived in the Concise Guide to PHARMACOLOGY 2015/16 (Alexander *et al.,* 2015a,b,c).

## Conflict of interest

Naveed Sattar has consulted for Amgen, Sanofi and is an investigator in the CANTOS trial. He was also on the steering committee for ENTRACTE. The other authors declare that the research was conducted in the absence of any commercial or financial relationships that could be construed as a potential conflict of interest.
